# Effectiveness of Project ECHO Programs in Improving Clinician Knowledge and Confidence in Managing Complex Psychiatric Patients: a Waitlist-Controlled Study

**DOI:** 10.1007/s40596-022-01701-5

**Published:** 2022-09-09

**Authors:** Erica Bessell, Ji Sun Kim, Lyn Chiem, Andrew McDonald, David Thompson, Nicholas Glozier, Andrew Simpson, Lisa Parcsi, Richard Morris, Rebecca Koncz

**Affiliations:** 1 The University of Sydney, Sydney, NSW Australia; 2 Sydney Local Health District, Sydney, NSW Australia

**Keywords:** Project ECHO, Eating disorders, Intellectual disability mental health, Continuing professional development, Teleconferencing

## Abstract

**Objective:**

The authors aimed to determine if Project Extension for Community Healthcare Outcomes (ECHO), a health-education model utilising teleconferencing technology, improves the capacity of clinicians in assessing and managing complex psychiatric patients.

**Methods:**

Three pilot Project ECHO programs were evaluated as a prospective waitlist-controlled trial, focusing on Adult Eating Disorders, Adult Intellectual Disability Mental Health, and General Mental Health. Each program comprised 9–10 weekly teleconferencing group sessions. Participants and waitlist-controls completed pre- and post-program surveys. The primary outcomes were self-reported knowledge and confidence in assessing and managing complex patients relevant to each group. Linear mixed models were used to assess the group-by-time interaction, or change over time, as appropriate.

**Results:**

Between July 2020 and June 2021, three series of the Adult Intellectual Disability Mental Health program, two series of the Adult Eating Disorders program, and two series of the General Mental Health program were delivered. Compared to waitlist-controls (*n* = 21), there were statistically significant improvements in self-reported knowledge and confidence for all topics amongst participants of the Adult Eating Disorders program (*n* = 44). In the Adult Intellectual Disability Mental Health program, there were significant improvements in self-reported knowledge and confidence amongst participants (*n* = 67) for most topics compared to controls (*n* = 21). There were no waitlist-controls for the General Mental Health program, but within-group analysis (*n* = 28) showed significant improvements in participants’ knowledge and confidence following program completion, compared to baseline.

**Conclusion:**

Project ECHO is a feasible and effective model to develop workforce capacity in managing complex psychiatric conditions.

The mental health needs of adults with complex disorders are frequently under-recognised and undertreated, including those with an eating disorder [1] and those with an intellectual disability [2]. Additionally, people who live with severe mental illness experience high rates of physical health comorbidity and early mortality [3, 4]. Recent national and state-level government mental health plans have called for improvements in the Australian mental health workforce capacity to allow quality care to be delivered equitably by more services [5, 6].

Eating disorders are serious and complex mental disorders associated with a high level of morbidity and mortality as well as significant socio-economic costs [7]. According to a gap analysis, only 22% of people with an eating disorder have access to specialist treatment [1]. Further to this, there is no systematic approach embedding eating disorder treatment into mainstream health systems with the majority of people seeking treatment experiencing difficulty getting access to appropriate treatment [1]. From a workforce perspective, up to 97% of clinicians surveyed had received no or insufficient training in eating disorders to enable them to provide treatment with confidence [1]. Thus, improving access to and quality of treatment for people with eating disorders is vital.

Similarly, adults living with an intellectual disability have substantial unmet mental health needs. Close to half a million people in Australia are living with an intellectual disability, and it is estimated that more than half of this population have a comorbid psychiatric disorder [2]. Despite these increased rates of psychiatric illness, a 14-year longitudinal cohort study demonstrated that only 10% of those will access adequate mental health care [8]. Limited knowledge and skills of hospital-based staff are commonly cited contributors to these unmet patient needs [9]. Furthermore, mental health professionals report low confidence in working with adults with intellectual disability, associated with inadequate education and training [10]. Thus, developing a skilled workforce is imperative to improve the quality of and access to mental health services for people with intellectual disability [11].

According to the 2007 National Survey of Mental Health and Wellbeing, 45% of adult Australians had experienced a mental disorder at some point in their lives, including 20% (3.2 million) who had experienced a mental disorder in the last 12 months [12]. In addition to this, 80% of people with a diagnosis of mental illness also have at least one co-existing chronic physical health condition [12]. People who live with a severe and enduring mental illness have a 13–30-year shorter life expectancy than the general population [3]. This is largely attributed to high rates of modifiable cardiometabolic risk factors such as smoking, hyperlipidaemia, hypertension, obesity, poor diet, and sedentary lifestyle, which translates to high rates of chronic disease [4]. Thus, improving the physical health of people living with mental illness to reduce early mortality is a national priority area [5].

Project Extension for Community Healthcare Outcomes (ECHO) is an internationally recognised program developed by the University of New Mexico [13]. It is a health education program that utilises teleconferencing technology to increase the capacity of health care clinicians working in areas with limited access to specialist care. Project ECHO creates knowledge-sharing networks between expert specialist groups (at the ‘Hub’) and primary health care providers or local health care providers (at the ‘Spokes’) through teleECHO sessions. TeleECHO sessions consist of brief didactic lectures by specialists and de-identified case-based presentations by clinician participants. Through this model of education, experts at the Hub provide mentoring to clinicians and all participants learn from each other. The goals of Project ECHO are to use teleconferencing technology to leverage scarce health care resources, to share best practices and reduce variation in care to improve outcomes, and to develop speciality expertise amongst primary health care providers through case-based learning [14].

Project ECHO has been trialled in at least 59 countries in various settings [15]. Research has found that the ECHO model is effective in increasing primary health care providers’ capacity to provide specialised care traditionally delivered by specialists in hospital settings, across a variety of health conditions, including hepatitis C, diabetes, and chronic pain [16, 17]. Importantly, several previous studies have demonstrated the feasibility and utility of Project ECHO specifically in mental health care. For example, ECHO Ontario Mental Health, established in 2015 and arguably one of the world’s largest Hubs for ECHO mental health programs, has demonstrated implementation success in terms of program acceptability, appropriateness, adoption, and cost-effectiveness [18]. Several Project ECHO mental health programs have also significantly improved health care clinicians’ knowledge and self-efficacy, including in relation to general mental health [19], geriatric mental health [20], integrated physical and mental health care [21], and for mental health conditions in the context of autism spectrum disorder [22].

Sydney Local Health District (SLHD) launched three pilot programs for Project ECHO in the latter half of 2020 with two programs run by state-wide expert mental health teams specialising in Adult Eating Disorders and Adult Intellectual Disability Mental Health, as well as a General Mental Health program targeted at clinicians in primary care with an emphasis on cardiometabolic health. The use of the Project ECHO model is relatively new in the Australian context, thus evaluating the efficacy of these programs is an important contribution to the local evidence base. Furthermore, no other Australian ECHO program at present focuses on upskilling clinicians in the management of adults with complex mental health conditions. The current global pandemic and associated increase in the use of teleconferencing technology for clinician education also contributes to the timeliness of programs such as this. We aimed to evaluate whether these ECHO programs increased clinician knowledge of the speciality area and enhanced their confidence in providing specialist care directly to patients in their own practice.

## Methods

### Development and Implementation of the ECHO Programs

The three ECHO teams developed the curricula by identifying key learning needs of potential participants through needs assessment surveys. Each needs assessment survey included 20 potential topics that were developed by the team of specialist clinicians for each Hub and were distributed through relevant networks to reach the anticipated participants of each program. Survey respondents were asked for basic demographic information (profession and service setting), and then asked to rate their interest and confidence for each proposed topic based on a 5-point Likert scale — from ‘not at all interested’ to ‘very interested’, and from ‘not at all confident’ to ‘very confident’. The final didactic lecture topics thus reflected the learning needs of health care providers and health needs of their patients based on the topics that participants rated most frequently of high interest and low confidence, as reviewed and finalised by expert consensus amongst the Hub team members. The final didactic lecture topics for each ECHO program are available from the corresponding author on reasonable request. There were some minor changes between series, particularly for the Adult Intellectual Disability Mental Health program, to reflect evolving feedback.

#### Adult Eating Disorders

The Project ECHO Adult Eating Disorders program was run by the New South Wales (NSW) Eating Disorders Outreach Service, which provides clinical consultation and support, and education and training to government- or state-employed clinicians managing adults with eating disorders across NSW. The program was designed to increase the specialist knowledge and expertise in managing adults with eating disorders. The primary audience were government- or state-employed clinicians across NSW. The program was also open to primary health care providers who provide care to adults with eating disorders. In 2020, the program ran two series (1.5 hours per week, 10 weeks per series).

#### Adult Intellectual Disability Mental Health

The Project ECHO Adult Intellectual Disability Mental Health program was run by the Statewide Intellectual Disability Mental Health Outreach Service, which aims to support the provision of quality mental health care to adults with an intellectual disability and build clinician capacity throughout NSW. This program provided training opportunities to government- or state-employed clinicians, primary care, and the National Disability Insurance Scheme provider sector across NSW. The program ran two series in 2020 and one series in 2021 (1.5 hours per week, 9–10 weeks per series).

#### General Mental Health

The Project ECHO General Mental Health program was run by the SLHD Mental Health Service. The program aims to support primary health care providers to build capacity in the treatment and management of people with severe and enduring mental illness. There is a focus on cardiometabolic risk and complex psychopharmacology. This program targeted primary health care providers across NSW, but especially providers in inner Sydney who support people with mental health conditions. The program was particularly intended for general practitioners, practice nurses, and allied health clinicians from the primary care sector. The program ran one series in 2020 and one series in 2021 (1 hour per week, 10 weeks per series).

### Study Design and Participants

We implemented a prospective waitlist-controlled evaluation of the three Project ECHO programs. All participants provided informed consent. This study was approved by the SLHD Human Research Ethics Committee – Concord Repatriation General Hospital (2020/ETH00629).

Clinicians who registered to participate in a Project ECHO SLHD program during the period July 2020–June 2021 were invited to participate in the evaluation. To be eligible to participate in a program, a clinician must be a health professional who provides care to adults with an eating disorder, intellectual disability, or other complex mental health issues. This included government- or state-employed medical, nursing, and allied health staff; clinicians working in private practice including general practitioners; or clinicians working for other non-government organisations.

The first series of the Adult Eating Disorders and Adult Intellectual Disability Mental Health programs aimed to have ~20 participants in attendance at each session as a pilot. Given the demand for the programs, the target was thereafter increased to ~50 participants for subsequent series across all three programs. Waitlisted clinicians included those who had registered their interest for a program but were unable to participate due to group limits being reached or their personal unavailability. These waitlisted individuals were invited to form the waitlist control group. At the end of the waitlist period, they were offered to enrol in the next series of their preferred ECHO program. The potential size of the waitlist control group was thus dependent on the number of clinicians who registered their interest above the group limit for each series or who were unavailable to participate in the current series. The waitlisted clinicians were included in this evaluation as a control group to allow comparison between groups in real time. If the group limit was not reached, the intervention group was optimised and there was no control group. In this case, the analyses were limited to within-group pre- and post-intervention comparisons.

### Program Evaluation

The surveys were based on Moore’s widely accepted conceptual framework to evaluate continuing medical education, which has been used to evaluate Project ECHO programs internationally [23]. Moore’s framework consists of seven levels to evaluate the impact of continuing medical education programs. Based on the first four levels of Moore’s framework, this study evaluated the utility of Project ECHO SLHD programs by assessing the following aspects: (1) ECHO participation, (2) participant satisfaction, (3) knowledge, and (4) confidence/competence.

Surveys were administered to all ECHO participants and waitlist controls within the week prior to the commencement of a program (pre-survey) and re-administered for completion within 2 weeks of the program completion (post-survey). Surveys were developed by expert consensus for the respective Hubs and administered to participants online via REDCap [24]. The pre-surveys included questions relating to participant demographics, and self-reported knowledge and confidence on topics related to the ECHO program curriculum. The post-surveys included questions relating to program participation, satisfaction with the program, and self-reported knowledge and confidence on the same topics. Participants rated their level of knowledge and confidence on a 5-point Likert scale (very low, low, moderate, high, very high). The pre- and post-surveys are available from the corresponding author on reasonable request.

### Statistical Analyses

The data were analysed using Microsoft Excel 2016 (Redmond, WA, USA), SPSS Statistics version 26 (Chicago, DE, USA), and RStudio 1.4.1717 (Boston, MA, USA). Data on participation, participant satisfaction, and self-reported knowledge and confidence were pooled into one group for the two Adult Eating Disorders series, another group for the three Adult Intellectual Disability Mental Health series, and a third group for the two General Mental Health series, respectively, to optimise the sample size for the analyses for each program. The data are presented as frequencies with percentage (participant demographic and satisfaction variables) and means with standard deviation (knowledge and confidence variables). Comparisons were made for self-reported knowledge and confidence for each topic assessed in the surveys, as well as the mean of all topics. Linear mixed models (LMMs) were used to assess the group (ECHO participants vs. waitlist controls) by time interaction for the Adult Eating Disorders and Adult Intellectual Disability Mental Health programs. For the General Mental Health program, there were no waitlist controls so LMMs were used to assess the change scores amongst participants only. LMM accounts for the expected correlation between pre- and post-scores within subjects while also providing improved statistical power by using all data (including participants and controls who only completed one of the two surveys) [25]. A sub-analysis was completed, where the LMM analyses were repeated using just those participants and waitlist controls who completed both the pre- and post-program evaluation surveys and these results are available from the corresponding author on reasonable request. A *p*-value of < 0.05 was considered statistically significant.

## Results

### Project ECHO Participation

During the evaluation period (July 2020–June 2021), a total of 243 people registered for one of the Project ECHO SLHD programs. Of these, 40 people (16%) did not attend any sessions and 10 people (4%) withdrew during the program. The other 193 people (79%) were considered active participants for the duration of the program. There was a mean attendance rate of 71% per session. Table 1 details the participation for each program.

**Table 1:** Participation details for each Project ECHO program, as well as overall

Programs	Adult Eating Disorders	Adult Intellectual Disability Mental Health	General Mental Health	Total
Series	**2**	**3**	**2**	**7**
Registrations	68	123	52	**243**
Non-attendance^a^	7 (10%)	20 (16%)	13 (25%)	**40 (16%)**
Withdrawn^b^	5 (7%)	2 (2%)	3 (6%)	**10 (4%)**
Participants	**56 (82%)**	**101 (82%)**	**36 (69%)**	**193 (79%)**
Nurse	14 (25%)	17 (17%)	8 (22%)	39 (20%)
Psychologist	11 (20%)	17 (17%)	3 (8%)	31 (16%)
Medical practitioner^c^	4 (7%)	5 (5%)	**17 (47%)**	26 (13%)
Behavioural support practitioner	0 (0%)	**23 (23%)**	0 (0%)	23 (12%)
Social worker	4 (7%)	13 (13%)	3 (8%)	20 (10%)
Occupational therapist	4 (7%)	13 (13%)	0 (0%)	17 (9%)
Dietitian	**17 (30%)**	0 (0%)	0 (0%)	17 (9%)
Other	2 (4%)	13 (13%)	5 (14%)	20 (10%)

^a^Non-attendance indicates those people who registered for the program, then did not attend any sessions

^b^Withdrawn participants were those people who started the program, then formally withdrew during the program

^c^Medical practitioners included psychiatrists, psychiatry trainees, rehabilitation physicians, and general practitioners

Amongst the 193 participants, 109 participants (56%) were government or state employees and 84 participants (44%) worked in general practices, private practices, non-government services, and the National Disability Insurance Scheme sector. There was strong penetration throughout NSW, with 118 participants working in metropolitan areas, 74 participants working in rural and regional NSW, and one participant working in a state-wide role.

The professions represented in each program (Table 1) reflect the differences in speciality areas and target audiences. Participants of the Adult Eating Disorders program were mainly dietitians (30%), nurses (25%), and psychologists (20%). Participants of the Adult Intellectual Disability Mental Health program included behavioural support practitioners (23%), nurses (17%), psychologists (17%), social workers (13%), and occupational therapists (13%). Participants of the General Mental Health program were mainly general practitioners (47%) and nurses (22%). Overall, nurses and psychologists represented the greatest proportion of participants in the ECHO programs (20% and 16% respectively).

For the Adult Eating Disorders program and the Adult Intellectual Disability Mental Health program, there were no statistically significant differences in age, profession, primary practice location, or previous relevant training between the ECHO participants and the respective waitlist controls (detailed results are available from the corresponding author on reasonable request). There were no waitlist controls for the General Mental Health program.

### Participant Knowledge and Confidence

Of the 56 ECHO participants of the Adult Eating Disorders program, 42 (75%) responded to the pre-program evaluation survey and 24 (43%) responded to the post-program evaluation, with a total of 44 participants included in the LMM. Compared to waitlist controls (*n* = 21), participants of the Adult Eating Disorders program reported a statistically significant improvement in knowledge and confidence for all seven topics assessed (Table 2). We found no differences in the demographic characteristics or pre-program knowledge and confidence ratings between those who completed both the pre- and post-surveys and those who completed only the pre-survey. Furthermore, in the sub-analysis including only those participants and waitlist controls who completed both surveys, statistically significant improvements in participants’ knowledge and confidence compared to waitlist controls were found for all but one of the topics assessed; this topic being ‘common comorbidities’. Detailed results are available on reasonable request from the corresponding author.

**Table 2:** Self-reported knowledge and confidence amongst the participants (*n* = 44) and waitlist controls (*n* = 21) for the Project ECHO Adult Eating Disorder program

	Knowledge^a^					Confidence^a^				
	Participants^b^		Waitlist controls^b^		Difference in change between groups^c^	Participants^b^		Waitlist controls^b^		Difference in change between groups^c^
	Pre	Post	Pre	Post		Pre	Post	Pre	Post	
Assessment	2.8 (0.9)	3.8 (0.6)	2.8 (1.1)	2.2 (1.1)	1.5 (0.8, 1.9) *	2.6 (0.9)	3.5 (0.5)	2.5 (1.0)	2.2 (1.1)	1.2 (0.4, 1.7) *
Medical and psychological complications	2.8 (0.9)	3.7 (0.6)	2.8 (1.2)	2.0 (1.0)	1.7 (0.8, 2.2) *	2.6 (0.9)	3.5 (0.7)	2.5 (1.0)	2.2 (1.1)	1.3 (0.4, 1.6) *
Common comorbidities	2.9 (0.8)	3.6 (0.7)	2.8 (1.1)	2.4 (1.3)	1.1 (0.2, 1.7) *	2.6 (0.9)	3.4 (0.7)	2.7 (1.0)	2.2 (1.3)	1.2 (0.2, 1.7) *
Managing challenging behaviour	2.5 (0.9)	3.5 (0.7)	2.3 (0.9)	2.0 (1.0)	1.3 (0.4, 2.0) *	2.5 (0.9)	3.4 (0.7)	2.2 (0.8)	1.8 (0.8)	1.4 (0.7, 2.0) *
Developing a collaborative treatment plan	2.6 (1.0)	3.8 (0.7)	2.4 (0.9)	2.2 (1.1)	1.3 (0.5, 2.0) *	2.5 (0.9)	3.5 (0.8)	2.4 (0.9)	2.2 (1.1)	1.3 (0.7, 1.8) *
Working with the family/carer	2.5 (0.9)	3.5 (0.7)	2.2 (0.7)	1.8 (0.8)	1.5 (0.6, 2.2) *	2.4 (0.9)	3.3 (0.6)	2.0 (0.7)	2.0 (0.7)	1.0 (0.3, 1.6) *
Navigating the use of the Mental Health Act	2.2 (0.9)	3.2 (0.8)	2.0 (0.9)	1.8 (0.8)	1.3 (0.5, 1.9) *	2.0 (0.8)	3.0 (0.8)	1.9 (0.8)	1.6 (0.5)	1.3 (0.7, 1.7) *
**Mean of all topics**	2.6 (0.8)	3.6 (0.6)	2.5 (0.8)	2.1 (1.0)	1.4 (0.7, 1.8) *	2.5 (0.8)	3.4 (0.6)	2.3 (0.8)	2.0 (0.9)	1.2 (0.6, 1.6) *

^a^Knowledge and confidence were rated on a 5-point Likert scale (1 = very low, 2 = low, 3 = moderate, 4 = high, 5 = very high)

^b^Pre- and post-scores are mean (standard deviation)

^c^The ‘difference in change’ is the difference in the change score between groups (Δ ECHO participants − Δ controls), reported as the mean difference (95% confidence interval)

*p < 0.05 for the between-group comparison (participants vs. waitlist controls) using linear mixed modelling

In the Adult Intellectual Disability Mental Health program, of the 101 participants, 59 (58%) responded to the pre-program survey and 40 (40%) responded to the post-program survey, with a total of 67 participants included in the LMM. Compared to waitlist controls (*n* = 21), participants reported statistically significant improvements in nine of twelve topics for knowledge and in eight of twelve topics for confidence (Table 3). Those who completed both the pre- and post-surveys rated their pre-program knowledge in one of the twelve topics (‘co-occurring developmental disabilities’) statistically significantly higher than those who only completed the pre-survey. No other statistically significant differences were identified. In the sub-analysis of those who completed both surveys, participants reported statistically significant improvements compared to controls in five of twelve topics for knowledge (‘screening and assessment’, ‘mood and anxiety disorders’, ‘challenging behaviour’, ‘sexuality and sexual behaviours of concern’, and ‘navigating the National Disability Insurance Scheme’) and in seven of twelve topics for confidence (‘screening and assessment’, ‘mood and anxiety disorders’, ‘psychosis’, ‘awareness of physical health comorbidities’, ‘challenging behaviour’, ‘sexuality and sexual behaviours of concern’, and ‘co-occurring development disabilities’). Detailed results are available from the corresponding author on reasonable request.

**Table 3:** Self-reported knowledge and confidence in participants (*n* = 67) and waitlist controls (*n* = 21) for the Project ECHO Adult Intellectual Disability Mental Health program

	Knowledge^a^					Confidence^a^				
	Participants^b^		Waitlist controls^b^		Difference in change between groups^c^	Participants^b^		Waitlist controls^b^		Difference in change between groups^c^
	Pre	Post	Pre	Post		Pre	Post	Pre	Post	
Screening and assessment	2.6 (0.8)	3.5 (0.7)	2.4 (0.7)	2.2 (0.8)	1.1 (0.5, 1.8) *	2.4 (0.8)	3.3 (0.6)	2.3 (1.0)	2.3 (0.7)	0.8 (0.3, 1.5) *
Mood and anxiety disorders	2.7 (0.6)	3.6 (0.6)	2.9 (0.7)	2.7 (0.9)	1.1 (0.7, 1.7) *	2.7 (0.7)	3.5 (0.7)	2.6 (0.9)	2.9 (1.2)	0.5 (0.1, 1.2) *
Psychosis	2.5 (0.7)	3.3 (0.7)	2.9 (0.7)	2.8 (1.1)	0.9 (0.2, 1.5) *	2.5 (0.8)	3.3 (0.7)	2.7 (0.8)	2.8 (1.0)	0.7 (0.2, 1.5) *
Evaluating risk and crisis management	3.0 (0.7)	3.7 (0.7)	3.2 (0.9)	3.0 (1.1)	0.9 (0.3, 1.5) *	2.9 (0.7)	3.7 (0.8)	2.8 (0.8)	2.9 (1.1)	0.7 (0.1, 1.3) *
Awareness of physical health comorbidities	3.0 (0.7)	3.8 (0.7)	3.1 (0.8)	3.0 (0.7)	0.9 (0.3, 1.5) *	2.9 (0.7)	3.8 (0.7)	2.7 (0.9)	2.8 (0.4)	0.9 (0.3, 1.4) *
Challenging behaviour	3.2 (0.7)	3.9 (0.8)	3.2 (1.0)	3.3 (0.9)	0.6 (0.0, 1.3) *	3.0 (0.7)	3.8 (0.7)	2.9 (0.9)	3.0 (1.1)	0.7 (0.2, 1.5) *
Role of psychotropic medication use	2.7 (0.7)	3.5 (0.8)	3.0 (1.1)	3.4 (1.0)	0.3 (−0.2, 1.1)	2.4 (0.9)	3.4 (0.9)	2.7 (1.0)	3.1 (1.3)	0.6 (−0.1, 1.5)
Psychological interventions	2.7 (0.6)	3.6 (0.7)	2.3 (0.9)	3.0 (1.0)	0.2 (−0.3, 0.9)	2.7 (0.7)	3.5 (0.7)	2.3 (0.9)	3.0 (1.0)	0.1 (−0.4, 0.9)
Sexuality and sexual behaviours of concern	2.5 (0.7)	3.4 (0.6)	2.7 (1.0)	2.6 (0.7)	1.0 (0.5, 1.7) *	2.3 (0.7)	3.3 (0.6)	2.5 (0.7)	2.7 (0.9)	0.8 (0.3, 1.5) *
Co-occurring developmental disabilities	2.6 (0.7)	3.6 (0.7)	2.7 (1.0)	2.8 (1.0)	0.8 (0.1, 1.4) *	2.6 (0.7)	3.6 (0.7)	2.5 (0.8)	2.7 (0.9)	0.9 (0.3, 1.5) *
Navigating the National Disability Insurance Scheme	2.9 (0.8)	3.5 (0.8)	3.0 (1.3)	2.6 (1.0)	1.0 (0.4, 1.5) *	2.9 (0.8)	3.5 (0.9)	2.6 (1.1)	2.4 (1.2)	0.7 (−0.0, 1.2)
Supporting carers	3.0 (0.8)	3.7 (0.8)	2.9 (0.9)	3.1 (0.9)	0.5 (−0.0, 1.1)	3.0 (0.8)	3.7 (0.8)	2.7 (1.0)	3.0 (1.0)	0.4 (−1.0, 0.3)
**Mean of all topics**	2.8 (0.4)	3.6 (0.5)	2.9 (0.7)	2.9 (0.6)	0.8 (0.4, 1.2) *	2.7 (0.5)	3.5 (0.5)	2.6 (0.7)	2.8 (0.8)	0.7 (0.4, 1.2) *

^a^Knowledge and confidence were rated on a 5-point Likert scale (1 = very low, 2 = low, 3 = moderate, 4 = high, 5 = very high)

^b^Data are mean (standard deviation)

^c^The ‘difference in change’ is the difference in the change score between groups (Δ ECHO participants − Δ controls), reported as the mean difference (95% confidence interval)

*p < 0.05 for the between-group comparison (participants vs. waitlist controls) using linear mixed modelling

There were no waitlist controls for the General Mental Health program; thus, the LMM only compared pre-program responses (*n* = 26) with post-program responses (*n* = 16) (Table 4). Statistically significant improvements were identified in nine of the ten topics assessed for self-reported knowledge, and in all topics assessed for self-reported confidence. In the sub-analysis including only the 12 participants who completed both surveys, statistically significant improvements were seen in three topics for knowledge (‘suicide prevention interventions’, ‘weight gain and mental health’, and ‘psychotropics and cardiometabolic effects’) and in eight topics for confidence (all topics except for ‘urgent mental health assessment’ and ‘treatment and management strategies for anxiety disorders in primary care settings’). It should be noted that between participants who completed both surveys and participants who only completed the pre-survey, there were statistically significant pre-program differences in three topics for self-reported knowledge (‘urgent mental health assessment’, ‘sleep disorders and mental health’, and ‘treatment and management strategies for anxiety disorders in primary care settings’) and one topic for self-reported confidence (‘urgent mental health assessment’). For each of these differences, pre-program ratings were higher in those who completed both surveys compared to those who only completed the pre-survey. Detailed results are available from the corresponding author on reasonable request.

**Table 4:** Self-reported knowledge and confidence amongst the participants (*n* = 28) of the Project ECHO General Mental Health program

	Knowledge^a^			Confidence^a^		
	Pre-program	Post-program	Change	Pre-program	Post-program	Change
Urgent mental health assessment	3.2 (0.8)	3.9 (0.7)	0.4 (0.6) *	2.8 (0.8)	3.6 (0.7)	0.8 (0.8) *
Diagnosis and intervention for personality disorders in primary care settings	2.7 (0.8)	3.3 (0.8)	0.6 (0.8) *	2.4 (0.7)	3.2 (0.8)	0.8 (0.7) *
Sleep disorders & mental health	2.8 (0.8)	3.7 (0.8)	0.8 (0.7) *	2.6 (0.8)	3.6 (0.6)	0.9 (0.6) *
Suicide prevention interventions	3.1 (0.8)	3.9 (0.7)	0.8 (0.7) *	2.7 (0.8)	3.6 (0.7)	0.9 (0.8) *
Treatment and management strategies for anxiety disorders in primary care settings	3.1 (0.8)	3.7 (0.9)	0.3 (0.5)	2.8 (0.8)	3.6 (0.8)	0.6 (0.6) *
Weight gain & mental health	3.2 (0.8)	3.8 (0.7)	0.6 (0.6) *	2.9 (0.7)	3.6 (0.6)	0.8 (0.6) *
Behaviour change interventions for depression	3.0 (0.8)	3.6 (0.7)	0.5 (0.6) *	2.7 (0.6)	3.6 (0.6)	0.8 (0.5) *
Treatment resistant depression	2.5 (0.8)	3.4 (0.8)	0.7 (0.7) *	2.2 (0.7)	3.2 (0.8)	1.0 (0.6) *
Psychotropics and cardiometabolic effects	3.1 (1.1)	3.6 (0.7)	0.5 (0.6) *	2.9 (0.9)	3.5 (0.6)	0.5 (0.6) *
Drug & alcohol use in people with enduring mental illness	3.0 (0.9)	3.6 (0.9)	0.5 (0.7) *	2.6 (0.8)	3.7 (0.8)	1.0 (0.5) *
**Mean of all topics**	3.0 (0.6)	3.6 (0.6)	0.5 (0.4) *	2.6 (0.5)	3.5 (0.6)	0.8 (0.4) *

^a^Knowledge and confidence were rated on a 5-point Likert scale (1 = very low, 2 = low, 3 = moderate, 4 = high, 5 = very high). Data are mean (standard deviation)

*p < 0.05 for the within-group comparison (pre- vs. post-program) using linear mixed modelling

### Participant Satisfaction

Across the three programs, there was a high level of satisfaction amongst the participants who responded to our post-program surveys (*n* = 80). Of these, 94% agreed that the didactic lectures increased their knowledge and that the case presentations were a helpful learning experience. Seventy-seven participants (96%) thought that the community of practice provided by the ECHO groups was helpful, and 88% agreed that participation in an ECHO program improved their access to specialist clinicians. Additionally, 94% of respondents agreed that they would recommend the program to other clinicians. Full details of participant satisfaction ratings for each program are in Table 5.

**Table 5:** Participant satisfaction ^a^ with the Project ECHO programs

	Adult Eating Disorders (*n* = 24)			Adult Intellectual Disability Mental Health (*n* = 40)			General Mental Health (*n* = 16)		
	Disagree	Neutral	Agree	Disagree	Neutral	Agree	Disagree	Neutral	Agree
ECHO improved my access to specialist clinicians	1 (4%)	3 (13%)	20 (83%)	0 (0%)	2 (5%)	38 (95%)	3 (19%)	1 (6%)	12 (75%)
The community of practice provided by the ECHO groups was helpful	0 (0%)	1 (4%)	23 (96%)	0 (0%)	1 (3%)	39 (98%)	1 (6%)	0 (0%)	15 (94%)
The technology was easy to use	0 (0%)	0 (0%)	24 (100%)	0 (0%)	1 (3%)	39 (98%)	0 (0%)	0 (0%)	16 (100%)
The technology worked well	0 (0%)	1 (4%)	23 (96%)	0 (0%)	3 (8%)	37 (93%)	0 (0%)	0 (0%)	16 (100%)
The topics presented throughout the curriculum increased my knowledge	0 (0%)	0 (0%)	24 (100%)	0 (0%)	4 (10%)	36 (90%)	0 (0%)	1 (6%)	15 (94%)
The case presentations were a helpful learning experience	0 (0%)	1 (4%)	23 (96%)	1 (3%)	3 (8%)	36 (90%)	0 (0%)	0 (0%)	16 (100%)
Overall, I have developed significant knowledge working with the target patient group	0 (0%)	3 (13%)	21 (88%)	0 (0%)	8 (20%)	32 (80%)	1 (6%)	0 (0%)	15 (94%)
Overall, I have significantly developed my confidence working with the target patient group	0 (0%)	2 (8%)	22 (92%)	0 (0%)	8 (20%)	32 (80%)	1 (6%)	1 (6%)	14 (88%)
I would recommend this program to other clinicians	0 (0%)	1 (4%)	23 (96%)	0 (0%)	4 (10%)	36 (90%)	0 (0%)	0 (0%)	16 (100%)

^a^Participants rated their agreement with each statement on a 5-point Likert scale. Two ratings indicating agreeance and two ratings indicating dis-agreeance were combined for the above

## Discussion

The current study demonstrates the feasibility and utility of Project ECHO programs for clinicians working with adults with complex psychiatric comorbidities. Participants of diverse professional backgrounds reported significantly increased knowledge and confidence in working with their respective cohorts; these findings were replicated in participants working with clinically distinct patients and made in comparison to waitlist-control groups where available. A range of professions and service settings were represented. The ECHO programs were overall well received, with a high level of satisfaction recorded.

The results of the pilot Adult Eating Disorders program support the utility of the ECHO model in the delivery of the NSW Eating Disorders Outreach Service. To date, there has only been one other publication reporting on the use of the ECHO model in upskilling clinicians in the assessment and management of patients with eating disorders [26]. Similar to the results of our evaluation, content analysis of participant feedback related to this program, implemented in western New York, USA, found increased participant knowledge and intended practice changes related to interdisciplinary teamwork, specialty-based practice, and early identification and intervention [26].

Likewise, the results of the pilot Adult Intellectual Disability Mental Health program support the utility of the ECHO model in addressing some of the gaps in the health inequity experienced by adults with an intellectual disability [10, 11], including access to specialist clinical advice regarding assessment and management. There is at least one other ECHO program globally specifically aimed at increasing the knowledge and confidence of clinicians treating adults with intellectual disability and comorbid mental health issues (ECHO Ontario); however, to our knowledge, there have been no other evaluations of ECHO programs targeted at this particular cohort. Previous ECHO programs focused on autism in children [27–29], and in teenagers and young adults [30], have included one or two sessions on mental health; however, upskilling clinicians in the mental health concerns of people with intellectual disability has not been the specific focus of any other published ECHO program. In this inaugural evaluation of an Adult Intellectual Disability Mental Health program, the ECHO model was found to increase participants’ access to specialist clinicians, and increased participants’ knowledge and confidence in most topics compared to waitlist controls.

There were no waitlist controls for the two series of the General Mental Health program, and the pre-post comparisons were limited by the low number of responses to the post-program survey and as such, limit the applicability of these findings. Previous publications of ECHO programs targeted at primary care clinicians in India [31, 32] and Canada [19] have reported improvements in participants’ knowledge and self-efficacy in treating patients with mental health issues; however, these programs did not emphasise cardiometabolic health in their lecture topics. Enduring mental illness has associated complexities in the treatment and management of both mental and physical health comorbidities. Many people with mental illnesses are entirely under the care of their general practitioner [33]; thus, evaluating ECHO programs which particularly target primary care clinicians, such as general practitioners, practice nurses, and allied health clinicians, is an important focus for future research.

There are several limitations to the current evaluation which should be considered. Firstly, it should be stated from the outset that the sample size for each group is relatively small and so results should be interpreted with caution. This is reflective of a particularly low response rate to the post-program surveys, although completers and non-completers did not significantly differ, and sub-analyses were performed which largely supported the primary results. This degree of attrition is in keeping with what could be expected with respect to longitudinal studies [34]. Moreover, compared to ECHO participants, fewer waitlist controls completed the surveys at both timepoints. ECHO participants and waitlist-controls were not instructed to avoid participation in other programs, although participation in comparable programs would theoretically confound these findings. However, given the paucity of ECHO programs available even internationally in each of these specialised areas, it is unlikely that participants or waitlist-controls would have participated in similar programs, and as such, the risk of confounding would be low.

Secondly, the pre- and post-program evaluation surveys used to evaluate the efficacy of these pilot programs have not been validated. Given the absence of existing standardised tools in the literature that were adaptable, the surveys were developed by expert consensus specific to the curriculum content and our study aims. Though statistically significant improvements were observed in participants’ self-reported knowledge and confidence compared to waitlist controls, as rated on a 5-point Likert scale, the magnitude of the clinical significance of these changes requires further evaluation. Thirdly, the evaluation of these pilot ECHO programs was limited to the first four levels of Moore’s framework, that is, participation, satisfaction, knowledge, and confidence/competence [23]. Furthermore, the assessment of changes in knowledge and confidence was limited to self-report and was assessed directly after participants completed the program. Thus, our assessment of the effectiveness of the Project ECHO programs is subjective in nature and does not include whether these improvements were sustained longitudinally.

Future evaluations of Project ECHO programs should focus on objective assessment of knowledge and confidence, as well as the higher levels of Moore’s framework, including behaviour change amongst clinician participants, patient-level outcomes, and system- or community-level outcomes [23]. Globally, evaluations of Project ECHO programs to these levels have been limited, and there have been calls for research teams implementing future ECHO programs to particularly focus on evaluating these levels to strengthen the evidence base [35].

Nonetheless, the success of these pilot programs supports the Project ECHO model as a potential tool for increasing the capacity of the workforce to care for people with complex mental health needs in community-based settings, reducing the number of people being undertreated and/or alleviating the potential bottleneck of referrals to specialist services. Identifying effective methods such as these to improve care will strengthen the response to the increasing demands for mental health care in Australia and beyond. Overall, the findings are in keeping with the success of Project ECHO programs internationally [16, 17]. The importance of these findings is particularly timely, as the pressures of a global pandemic have forced the education and health sectors to transition online, which fits well with the ECHO adage, ‘moving knowledge, not people’ [36]. Furthermore, nearly all participants across the three programs found the community of practice helpful, which highlights the advantages of the ECHO ‘all teach, all learn’ philosophy, and the value of ECHO case discussion in building expertise [37]. Taken together, programs delivered in the Project ECHO model have the potential to build capacity of frontline workers in delivering high-quality mental health care to patients with a diverse range of needs in a variety of settings, including in under-resourced rural and remote locations.

In conclusion, these pilot programs demonstrate the feasibility for Project ECHO implementation in managing complex psychiatric conditions. Based on these promising results, it would be reasonable to consider that the Project ECHO implementation could be transferable to a broad range of psychiatric conditions, with the aim of providing education and training to clinicians in the management of adults with complex mental disorders. This may be particularly relevant now during a global pandemic and beyond, as health education is having to fast adapt to online mediums. Our findings suggest that Project ECHO is implementable and a model conducive to capacity building even in this challenging context.
